# Cytokine Storm as a Cellular Response to dsDNA Breaks: A New Proposal

**DOI:** 10.3389/fimmu.2021.622738

**Published:** 2021-02-01

**Authors:** Snehal Shabrish, Indraneel Mittra

**Affiliations:** Translational Research Laboratory, Advanced Centre for Treatment, Research and Education in Cancer, Tata Memorial Centre, and Homi Bhabha National Institute, Mumbai, India

**Keywords:** Cytokine storm, dsDNA breaks, COVID-19, inflammation, apoptosis, resveratrol, copper, free radicals

## Abstract

Pathogenesis of cytokine storm is poorly understood. In this article we propose a new mechanism and suggest innovative therapeutic avenues for its prevention. We have reported that particles of cell-free chromatin (cfCh) that are released from the billions of cells that die in the body everyday can illegitimately integrate into genomes of healthy cells to trigger dsDNA breaks. The latter leads to apoptosis and/or intense activation of inflammatory cytokines in the affected cells. We hypothesise that a similar phenomenon of dsDNA breaks and inflammation is involved in cytokine storm. The abundant cfCh particles that are released from dying host cells following viral/microbial invasion initiate a cascading effect of more cell death resulting in a vicious cycle of further DNA damage, apoptosis and hyper-inflammation which culminate in cytokine storm. We propose that this unrelenting vicious cycle of cellular DNA damage and cytokine storm may be the underlying cause of high mortality from severe COVID-19. We discuss results of our preclinical studies wherein we have shown that endotoxin induced cytokine storm in mice can be reversed by three different agents that have the ability to inactivate cfCh. These agents may be worthy of investigation in clinical trials to reduce mortality from COVID-19.

**Graphical Abstract:**
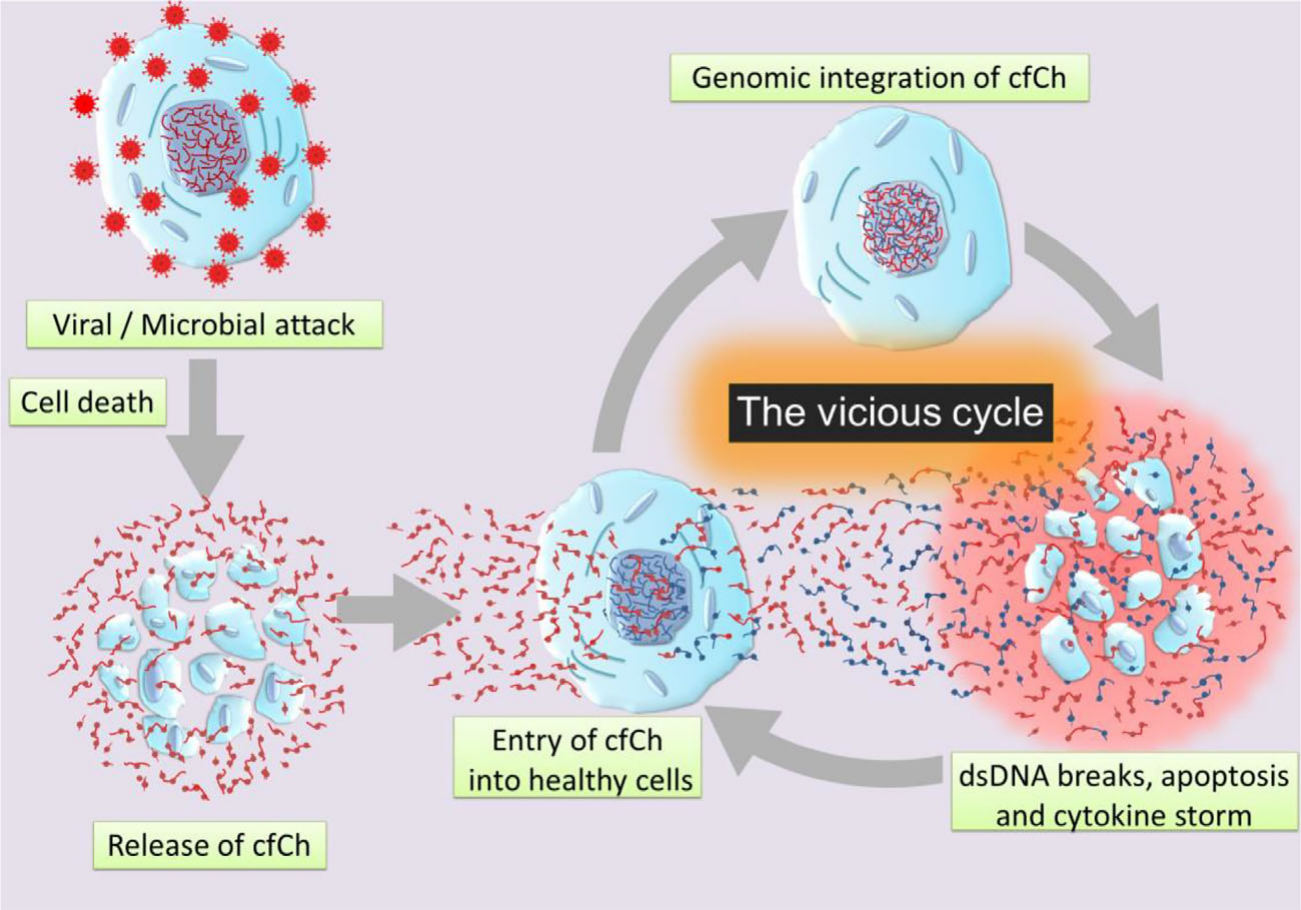
Schematic illustration of a vicious cycle initiated by genomic incorporation of cfCh resulting in dsDNA breaks, apoptosis, and hyper-inflammation which culminate in cytokine storm.

## Introduction

Cytokine storm is a condition characterized by an overwhelming and uncontrolled inflammation with major implications for global health (1). Cytokine storm is a critical component of the current COVID-19 pandemic, and is associated with severity of the disease and high mortality (2). In the worldwide flu pandemic of 1918, cytokine storm was a major cause of high death rate (3). Cytokine storm has also been described in other pandemics such as H1N1 swine flu (4), H5N1 avian flu (5) and severe acute respiratory syndrome coronavirus (SARS-CoV) (6). It is associated with sepsis in general which affects 48.9 million people worldwide every year of which 11 million die (7). Death from sepsis accounts for 19.7% of all global deaths, especially in poorer countries of the world (7). Several experimental studies and clinical trials have suggested that cytokine storm correlates directly with tissue injury, DNA damage and severity of the disease (1). In spite of intensive research, pathogenesis of the cytokine storm remains poorly understood, hindering development of effective therapies.

## Cytokine Storm: Summary of Current Knowledge

The innate immune response is activated by pattern recognition receptors (PRRs) in response to pathogen-associated molecular patterns (PAMPs) and/or damage-associated molecular patterns (DAMPs) (8). Activated immune response triggers intracellular signalling cascades in immune cells leading to production of inflammatory cytokines by various cells including macrophages, natural killer (NK) cells, dendritic cells, T cells, mast cells, endothelial and epithelial cells (8–10). The immune response is highly regulated and sequentially and temporally orchestrated (8). However, in certain pathological conditions, a profusion of PAMPs or DAMPs are released in response to cell death and stress (11–15). This causes hyper-stimulation of immune cells leading to intense secretion of inflammatory cytokines which results in the cytokine storm (13, 14). This hyper-inflammation triggering cytokine storm can either be in response to PAMPs which activates pathogen-induced hyper-inflammation, or to DAMPs which are self-molecules derived from host cells itself triggering auto-inflammatory response. Although it is widely accepted that these DAMPs and PAMPs are key molecules that trigger an inflammatory response (11–15), the precise nature of these molecules has not been characterized (16–18).

Recently, there has been a spurt of publications associating genomic stress and DNA damage in activation of inflammation (19–22). According to these reports, DNA that accumulates in the cytoplasm following DNA damage and/or microbial infection, acts as DAMPs and activates the DNA sensing GMP-AMP synthase-stimulator of interferon genes (cGAS-STING)-mediated pathway (19–22). The latter triggers an innate immune response by activating pro-inflammatory cytokines (19–22). In addition to microbial DNA and self-DNA from the nucleus, cGAS-STING pathway is also activated by cytosolic mitochondrial DNA (mtDNA) (23, 24). The latter has the potential to induce inflammatory responses and organ injuries in various diseases including cancer (25), diabetes (26), cardiovascular diseases (27) and trauma (28). Elevated levels of mtDNA in circulation has also been reported to be associated with severity of sepsis (29). Recent reports have also implicated presence of cytoplasmic chromatin fragments (CCF) in immune activation (30–32). CCF that are pinched off from nuclei during cellular senescence (33) are recognized by cGAS to stimulate an inflammatory response *via* STING (30–32). However, whether presence of DNA and/or CCF in the cytoplasm has the potential to trigger the cytokine storm or whether some other mechanism(s) is involved in triggering hyper-inflammation remains unknown. Thus, although the cytokine storm has been known for more than a century (1, 3), and much has been reported on its pathological consequences (1, 5), the trigger for the cytokine storm continues to remain elusive, hindering the development of effective therapies (34). Herein we put forward the hypothesis that cell-free chromatin (cfCh) particles (nucleosomes) released from dying host cells may contribute to the cytokine storm.

## Cell Free Chromatin (cfCh) as a Novel Trigger for the Cytokine Storm

### Origin and Structure of Cell-Free Chromatin (cfCh)

It has been estimated that 10^9^–10^12^ cells die in the body, primarily by apoptosis, every day (35). Apoptosis is characterized by nuclear and chromatin condensation followed by fragmentation of DNA by endogenous nucleases, especially caspase-3 and activated DNase (36). Although not demonstrated, it is likely that cfCh particles are also released following other forms of cell death such as necrosis, NETosis, pyroptosis (37). In spite of the body’s best efforts to get rid of cfCh (38, 39), a significant amount escapes into the extracellular compartments as well as into the blood circulation (40, 41). cfCh in circulation are cleared by the body by several mechanisms. These include: 1) phagocytosis by macrophages (42); 2) degradation by DNase I present in circulation (43), and 3) liver continuously removing cfCh resulting in a turnover half-life of 10–15 min (38, 39). Low baseline levels of cfCh in healthy individuals play a critical role in maintaining an efficient immune environment. However, elevated cfCh levels as seen in various clinical conditions may lead to runaway inflammation. These conditions have included autoimmune diseases (44), severe infections (45), trauma (46), burns (47), deep vein thrombosis (DVT) (48), cerebral stroke (49), malignancy (50). Increasing cfCh levels positively correlate with age (51).

### The Hypothesis

Our hypothesis is based on our recent finding that cfCh particles that are released from the hundreds of millions of cells that die in the body daily to enter into the blood stream can illegitimately integrate into genomes of healthy cells to damage their DNA by inducing dsDNA breaks (52, 53). Such events may also occur locally following cell death in tissues with release of cfCh which integrates into genomes of bystander cells in the neighbourhood (53, 54). Genomic integration of cfCh can have catastrophic consequences, especially since the DNA damage is repaired by the error-prone non-homologous end joining (NHEJ) mechanism, which further accentuates genomic mutations in the form of deletions, insertions, re-arrangements and chromosomal damage which may often cause apoptosis of the cells. The hypothesis also incorporates our recent observation that dsDNA breaks resulting from cfCh integration leads to intense activation of inflammatory cytokines (54, 55). Since cell death is markedly increased following viral or bacterial invasion, we hypothesise that illegitimate genomic integration of cfCh particles that are released from the dying host cells trigger a vicious cycle of more dsDNA breaks, apoptosis and hyper-inflammation which culminate in the cytokine storm (**Graphical Abstract**). We propose that the abundant cfCh that arise following viral/microbial invasion act as DAMPs and activate systemic inflammation. This proposal is supported by reports that circulating levels of cfCh are markedly elevated in patients admitted to ICU with severe sepsis (34). Since the latter is usually associated with the cytokine storm (56), it leads to the possibility that cfCh may be an important factor that contributes to the cytokine storm in sepsis.

### Can Cell-Free DNA and/or Free Histones Explain the Cytokine Storm?

Cell-free DNA (cfDNA) and free histones have been shown to have pro-inflammatory properties (57, 58). However, the immune stimulatory effects induced individually by cfDNA and free histones are different when compared to that induced when they are complexed in the form of cfCh (59). Furthermore, the question as to whether naked DNA and/or free histones are indeed present in circulation is in doubt. Apoptotic cell death results in chromosomal condensation and fragmentation with release of chromatin fragments and not of cfDNA or free histones (60). The existence of cfCh in serum and/or plasma can be easily detected by ELISA (61), while the demonstration of cfDNA requires DNA to be extracted from plasma/serum using Proteinase-K treatment. Therefore, the possibility that the isolated cfDNA has, in fact, been derived from circulating cfCh cannot be excluded. Reports of the existence of a direct and strong correlation between circulating cfCh and cfDNA would support such a possibility (62). Similarly, with respect to studies reporting immune-stimulatory effects of free histones (59, 63, 64), the methodologies used to quantify histones did not make a distinction between free histones and cfCh (59). Therefore, whether the latter are present in circulation also remains unclear (59). This uncertainty may have been put to rest by our recent observations made in relation to lipopolysaccharide (LPS) induced sepsis in a mouse model (65). Using confocal microscopy of histological sections of mouse vital organs after staining with fluorescent antibodies against DNA and histone H4, we have shown that it is cfCh, and not free DNA or histones, that are extruded from dying host cells following LPS treatment (65). Therefore, it is likely that cfCh, rather than cfDNA or free histones, is the agent responsible for initiating the cytokine storm in severe infection.

### cfCh in Circulation Integrate Into Genomes of Healthy Cells

Although existence of circulating cfCh particles has been known since 1990 (66), whether they have any patho-physiological role to play in the host has only recently been addressed (51, 52). Isolation of cfCh from sera of cancer patients followed by examination under electron microscope revealed particles of variable sizes (~10 nm >1000 nm) having a beads-on-a-string appearance characteristic of chromatin (52). When cfCh particles isolated from serum where fluorescently labelled and added to cultured mouse fibroblast cells, numerous cfCh particles could be detected in nuclei of recipient cells within 6h (52). The up-taken cfCh rapidly associated themselves with chromosomes of host cells which was followed by activation of an intense DNA damage repair response (DDR) followed by their incorporation into the host cell genomes (52). The activated DDR proteins included H2AX, ATR, ATM, P-p53, P-p21, MDC-1, GADD-34, RAD-50, NIBRIN, MRE-11, DNA-PKcs and DNA ligase IV (52). In addition, apoptotic pathway proteins namely, JC-1, cytochrome-C and caspase 3, were also activated (52) indicating that many of the affected cells were destined to undergo apoptotic cell death. Next generation sequencing detected tens of thousands of DNA reads of human origin in single cell clones developed from the cfCh treated mouse cells; while PCR amplification revealed presence of multiple human Alu sequences (52). cfCh integration resulted in dsDNA breaks as indicated by activation of H2AX which was seen both *in vitro* and *in vivo* (52). A unique mechanism was proposed by which cfCh particles integrate themselves into genomes of healthy cells, and which is facilitated by premature activation of DDR (discussed below).

### cfCh Released From Dying Cells Integrates Into Genomes of Bystander Cells

We have reported that cfCh released from dying host cells can also integrate into genomes of surrounding healthy bystander cells (54, 55). When human cancer cells were treated with ionizing radiation and co-cultured with mouse fibroblasts, human DNA (cfCh) signals could be detected in the nuclei of mouse cells by FISH (55). Confirmation that cfCh had actually integrated into the genomes was confirmed by detection of multiple human Alu sequences in the mouse cells (55). Bystander uptake and genomic integration of cfCh released from dying cells was also shown to occur in distant organs (55). When mice were delivered focused mini-beam irradiation (20 Gy) to the umbilical region, intense activation of H2AX, caspase 3, NFκB and IL-6 was detected in brain cells (55).

### cfCh Integrates Into the Genome by a Unique Mechanism

How does cfCh enter the cell and integrate themselves into the genome? Our microarray studies have revealed that pathways related to phagocytosis are maximally up-regulated as early as at 6h in mouse fibroblast cells in response to cfCh particles that are released from co-cultured dying Jurkat cells (54). This finding would suggest phagocytosis or pinocytosis to be one of the important mechanisms by which the cell ingests extraneous cfCh. Once inside the cell, cfCh particles integrate themselves into the genome of the host cell by a mechanism which is unique in being the opposite of the classical model of DNA damage and repair (52). According to the classical model of DNA damage, DDR is activated after the DNA damage occurs in response to agents such as ionizing and UV radiation and radiomimetic chemicals (67). According to the proposed new model, entry of cfCh into the cell misleads the cell into perceiving them as broken fragments of its own chromosomes containing dsDNA breaks at both ends (52). This prompts the cell to activate a premature DDR much before any damage to DNA having actually occurred. The activated DDR includes repair proteins such as DNA PKc, DNA ligase IV which link up the multiple heterogenous cfCh fragments into concatamers of different sizes. The latter, containing a mosaic of multiple discontinuous DNA segments in the form of conctamers, now form new substrates for incorporation into the genome of host cells, by non-homologous recombination (NHR). The resulting DNA damage is repaired by the error–prone NHEJ mechanism (68) which creates further mutations in the form of insertions, deletions, genetic rearrangements as well as chromosomal damage (52). Thus, paradoxically, DDR which is supposed to protect the integrity of the genome ends up damaging it by its premature activation. The formation of intracellular concatamers is supported by the argument that since the threshold for detection of FISH signals is of the order of 30–50 kilo bases (69), presence of human DNA signals in mouse cells detected by FISH indicates that relatively long human DNA sequences, rather than discrete cfCh particles, incorporate themselves into the mouse cell genomes. Genomic integration of cfCh concatemers by NHR leads to intense activation of inflammatory cytokines (discussed below).

### Genomic Integration of cfCh Leads to Somatic Mosaicism

Illegitimate genomic integration of cfCh, derived from the billions of cells that die in the body every day may result in dsDNA breaks and repair by NHEJ. These damaging events occurring repeatedly throughout life may generate multiple genomic polymorphisms which are likely to increase with age (53). Rapid and cumulative effects of DNA damage may exceed the adaptive capacity of the human genome in aging populations which leads to increased mutagenesis and development of various diseases, including cancer. This would be in accordance with the exploding literature fueled by advances in Next generation sequencing on the discovery of somatic mosaicism in healthy cells (70, 71). Somatic mosaicism is related to aging (72), cardiovascular diseases (73), Alzheimer’s disease (74) and cancer (75). The above discussion would suggest that approaches to retard aging would need to take into account accumulating dsDNA breaks that result from life-long and repeated genomic integration of cfCh.

### cfCh Integration, dsDNA Breaks, and Activation of Inflammatory Cytokines

**Figure 1:**
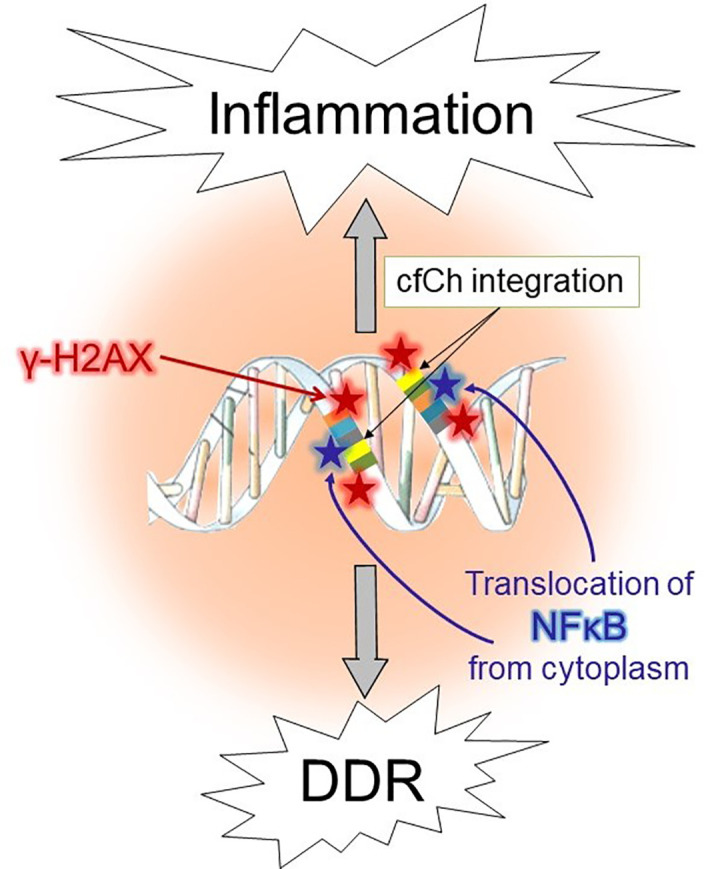
Schematic illustration of activation of DDR and inflammation following illegitimate integration of cfCh into the genome.

The aforementioned co-culture experiment of irradiated dying cancer cells of human origin with mouse fibroblasts, led to activation not only of H2AX but also of multiple inflammatory cytokines (54). The latter included NFκB, IL-6, TNF-α and IFN-γ, all of which were activated simultaneously by 6h (54), and their activation coincided with point of the maximal induction of H2AX (6h) (54). Co-activation of dsDNA breaks and inflammatory cytokines suggested an interrelationship between the two, which was further substantiated by microarray analysis which revealed activation of multiple pathways related to inflammation concurrently with those that accompany DNA damage and cell cycle at 6h (54). Injection of irradiated dying cancer cells pre-labelled with BrdU intravenously into mice led to uptake and genomic integration of BrdU labelled cfCh particles into nuclei of vital organs accompanied by activation of H2AX (54). Genomic integration of cfCh led to intense activation of multiple inflammatory cytokines to include NFκB, IL-6, TNF-α and IFN-γ. These experiments made the additional novel observation that fluorescent signals of γH2AX co-localized strictly with those of the transcription factor NFκB in the nuclei of vital organs (54). The inactivated form of NFκB normally remains sequestered in the cytoplasm (76) and trans-locates to the nucleus upon activation by stressful stimuli such as DNA damage (77). Although several nuclear translocation sites for NFκB have been described (78), the finding that γH2AX and NFκB fluorescence signals co-localize has led to the proposal that, following the catastrophic event of integration of cfCh into the genome and the consequent dsDNA breaks, NFκB is strongly activated, followed by its translocation from the cytoplasm to the sites of cfCh integration (79, 80). This finding indicated that inflammation might be a direct consequence of dsDNA breaks inflicted by integration of cfCh (80). It also suggests that cfCh acts as a major form of DAMPs. A schematic model to represent the relationship between cfCh induced dsDNA breaks and inflammation is given in **Figure 1**.

### Inactivation of cfCh Can Prevent the Cytokine Storm

We have identified several cfCh inactivating agents that can prevent the cytokine storm (55, 65, 81). These have included 1) anti-histone antibody complexed nanoparticles (CNPs) which inactivate cfCh by binding to histones; DNase I which inactivates cfCh by degrading its DNA component; and 3) a newly described pro-oxidant combination of the well-researched nutraceuticals Resveratrol and metallic Copper (R-Cu) which degrades cfCh through the medium of free radicals. We have recently reported that these cfCh inactivating/degrading agents can reverse the cytokine storm following endotoxin sepsis, chemotherapy and radiation therapy in mice. Details of these studies are given in the subsequent paragraphs.

### Inactivation of cfCh Can Prevent the Cytokine Storm in Endotoxin Sepsis

The International Sepsis Forum defines sepsis as “a life-threatening condition that arises when the body’s response to an infection injures its own tissues and organs” (82). This definition implies that hyper-inflammation and immune suppression in sepsis is a result of body’s own response against the pathogen and not due to the pathogen *per se* (83). We have recently shown in an endotoxin induced sepsis model that cfCh particles that are released from dying host cells following viral/microbial infection may be the agents that injure the body’s own tissues and organs that leads to sepsis - a finding which would be consistent with the above definition of the International Forum (65).

Several studies have reported that not only PAMPs, but also DAMPs, are recognized by pattern recognizing receptors (PRRs) expressed on immune-reactive cells (84–86). DAMPs are nuclear or cytoplasmic non-microbial molecules, released from the host cells following tissue injury which includes histones, cfDNA, chromatin, HMGB1, etc. (86). Clinical studies have shown a positive correlation of levels of DAMPs, especially of histones and nucleosomes, with sepsis severity (45, 87, 88).

**Figure 2:**
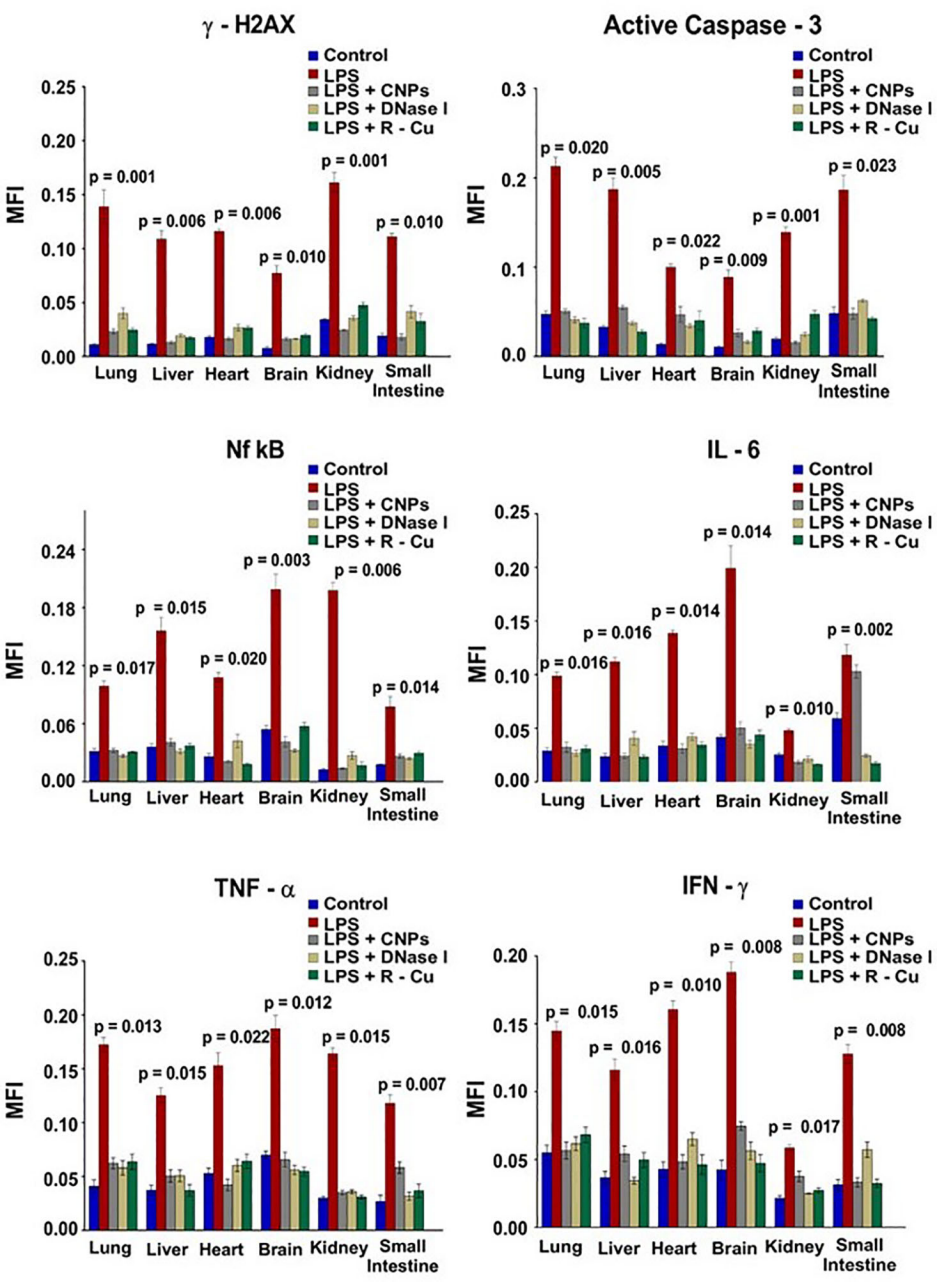
DNA damage, apoptosis and inflammation in multiple organs and tissues induced by LPS can be prevented by concurrent treatment with cfCh inactivating agents. The above parameters were estimated at 72hrs following LPS treatment by indirect immuno-fluorescence. Mean (± SEM) values between groups were compared using non parametric one-way ANOVA (Kurskal—Wallis test) with Dunn’s multiple comparison method at the significance and confidence level of p = 0.05. MFI = Mean fluorescence intensity. Reproduced from ref (65).

In our study, sepsis was induced in mice by injecting lipopolysaccharide (LPS), a bacterial membrane antigen, which led to extensive cell death and copious release of cfCh particles into extracellular spaces of vital organs and into the circulation (65) (**Figure 2**). cfCh particles thus released followed by their integration into genomes of surviving cells led to extensive dsDNA breaks and apoptosis in cells of multiple organs *viz.*, lung, liver, heart, brain, kidney and small intestine (65), as well as those of immune related organs such as thymus and spleen. cfCh integration and dsDNA breaks led to intense activation of inflammatory cytokines CRP, IL-6, IL-1β, TNF-α, and IFN-γ in multiple organs as well as release of these cytokines in circulation. The extensive DNA damage also led to immune suppression, coagulopathy, fibrinolysis, thrombocytopenia, multi- organ failure and death. All the above pathologies could be abrogated by administration of the cfCh inactivating agents to mice concurrently with LPS. This data provided strong evidence for a relationship between cfCh integration, dsDNA breaks, cytokine storm and sepsis.

### Inactivation of cfCh Can Prevent the Cytokine Storm Associated With Chemotherapy and Radiation Therapy

Cancer treatments involving chemotherapy and radiation therapy are known to trigger intense activation of pro-inflammatory cytokines (89, 90). The latter is thought to be activated by unidentified molecules which act as DAMPs and stimulate immune cells to release pro-inflammatory cytokines (91). However, the nature of these DAMPs continues to remain unidentified (17, 92). We have shown that, as in the case of sepsis, cfCh released from chemo- or radio therapy induced dying cells are the elusive DAMPs. Therapy induced cell death and cfCh release triggers a cascading effect of more cell death leading to a vicious cycle of further rounds of DNA damage, apoptosis and inflammation which perpetuate and amplify the toxic effects of these cancer therapies (55, 81). We have further reported that administration of the above three cfCh inactivating agents interrupted this vicious cycle thereby preventing the toxic effects of cancer treatment (55, 81). This reinforces the conclusion reached above, with respect to endotoxin sepsis, that copious release of cfCh particles following cell death resulting from chemotherapy and radiation therapy act as DAMPs to trigger the cytokine storm.

### Is Cell Free Chromatin Implicated in Pathogenesis of COVID-19?

Pathogenesis of COVID-19 is not well understood. The disease primarily affects the lungs leading to hypoxemic respiratory failure, secondary bacterial pneumonia and direct tissue damage. The disease is also associated with the cytokine storm with excessive release of inflammatory cytokines which can cause multi-organ damage (93). The other organs that are affected include heart, nerves, brain, vessels, kidneys and skin. We have already alluded to the potential role of cfCh in the cytokine storm, but, currently, literature on direct measurement of cfCh levels in COVID-19 patients is lacking. Elevated levels of cfCh in these patients is to be expected since sepsis forms a major manifestations of the SARS-CoV-2 viral infection (94), and there is abundant literature to show that cfCh levels are elevated in sepsis (45, 87, 88, 95). As the title of current article suggests, and discussed extensively above, the cytokine storm is a likely consequence of DNA and cellular damage inflicted by cfCh. We propose that cfCh induced tissue/organ damage can not only explain the aetiology of the cytokine storm, but also help to explain the multi-organ injury that is associated with COVID-19 as a direct consequence of cfCh induced cellular DNA damage.

## Conclusion and Future Prospects

In this article we have proposed that inflammation may be a direct consequence of dsDNA breaks inflicted by genomic integration of cfCh released from dying host cells, and that cfCh may be the key instigators of the cytokine storm (54, 80). cfCh particles released from dying host cells following viral/microbial infection may trigger a cascading effect of more host cell death leading to a vicious cycle of further rounds of DNA damage, apoptosis and inflammation which perpetuate and amplify the pathological effects of the offending agent culminating in the cytokine storm. Although, currently, literature on direct measurement of cfCh levels in COVID-19 patients is lacking, we hypothesise that the high mortality in severe COVID-19 may be due to the cytokine storm related sepsis. The latter being perpetuated by the vicious cycle triggered by profuse release of cfCh particles that result from Corona virus induced cell death. The implication of such a suggestion is that, while eliminating the virus may result in resolution of disease in asymptomatic or early symptomatic COVID-19 patients, once the vicious cycle sets in, elimination of the virus may not prevent death in patients with severe disease. Indeed, a recent study has reported that effects of the cytokine storm can persist for a long time after the virus has been eliminated from the body (96). Furthermore, the observation that elderly patients and those with underlying ageing related co-morbidities such as diabetes (97) and cardio-vascular diseases (98) are more prone to COVID-19 related complications, may be attributable to elevated levels of cfCh in these conditions (51, 99, 100). We propose that treatment of severe COVID-19 should include cfCh inactivating agents to prevent death, and that these agents are worthy of investigation in clinical trials in patients suffering from severe COVID-19.

## Data Availability Statement

The original contributions presented in the study are included in the article/supplementary material. Further inquiries can be directed to the corresponding author.

## Author Contributions

IM conceptualized and wrote the article. SS wrote the article and prepared the figures. All authors contributed to the article and approved the submitted version.

## Funding

This study was supported by the Department of Atomic Energy, Government of India, through its grant CTCTMC to Tata Memorial Centre awarded to IM.

## Conflict of Interest

The authors declare that the research was conducted in the absence of any commercial or financial relationships that could be construed as a potential conflict of interest.
